# Dismal Survival in COVID-19 Patients Requiring ECMO as Rescue Therapy after Corticosteroid Failure

**DOI:** 10.3390/jpm11111238

**Published:** 2021-11-22

**Authors:** Sebastian Voicu, Antoine Goury, Thomas Lacoste-Palasset, Isabelle Malissin, Lucie Fanet, Samar Souissi, Julia Busto, Vincent Legros, Laetitia Sutterlin, Giulia Naim, Aymen M’Rad, Adrien Pepin-Lehaleur, Nicolas Deye, Bruno Mourvillier, Bruno Mégarbane

**Affiliations:** 1 Department of Medical and Toxicological Critical Care, Lariboisière Hospital, INSERM UMRS-1144, Paris-University, 75010 Paris, France; sebastian.voicu@aphp.fr (S.V.); thomas.lacoste-palasset@aphp.fr (T.L.-P.); isabelle.malissin@aphp.fr (I.M.); lfanet@chu-reims.fr (L.F.); samar.souissi@gmail.com (S.S.); laetitia.sutterlin@aphp.fr (L.S.); giulia.naim@aphp.fr (G.N.); mrad.aymen@gmail.com (A.M.); Adrien.Pepin-lehaleur@aphp.fr (A.P.-L.); Nicolas.deye@aphp.fr (N.D.); 2 Medical Critical Care Department, Robert Debré University Hospital, 51100 Reims, France; gouryantoine@gmail.com (A.G.); jbusto@chu-reims.fr (J.B.); bmourvillier@chu-reims.fr (B.M.); 3 Surgical Critical Care Department, Maison Blanche University Hospital, 51100 Reims, France; vlegros@chu-reims.fr

**Keywords:** ARDS, COVID-19, corticosteroid, dexamethasone, ECMO, survival

## Abstract

**(1)** Background: COVID-19 may lead to refractory hypoxemia requiring venovenous extracorporeal membrane oxygenation (ECMO). Survival rate if ECMO is implemented as rescue therapy after corticosteroid failure is unknown. We aimed to investigate if ECMO implemented after failure of the full-recommended 10-day corticosteroid course can improve outcome. **(2)** Methods: We conducted a three-center cohort study including consecutive dexamethasone-treated COVID-19 patients requiring ECMO between 03/2020 and 05/2021. We compared survival at hospital discharge between patients implemented after (*ECMO-after* group) and before the end of the 10-day dexamethasone course (*ECMO-before* group). **(3)** Results: Forty patients (28M/12F; age, 57 years (51–62) (median (25th–75th percentiles)) were included, 28 (70%) in the *ECMO-before* and 12 (30%) in the *ECMO-after* group. In the *ECMO-before* group, 9/28 patients (32%) received the 6 mg/day dexamethasone regimen versus 12/12 (100%) in the *ECMO-after* group (*p* < 0.0001). The rest of the patients received an alternative dexamethasone regimen consisting of 20 mg/day during 5 days followed by 10 mg/day during 5 days. Patients in the *ECMO-before* group tended to be younger (57 years (51–59) versus 62 years (57–67), *p* = 0.053). In the *ECMO-after* group, no patient (0%) survived while 12 patients (43%) survived in the *ECMO-before* group (*p* = 0.007). **(4)** Conclusions: Survival is poor in COVID-19 patients requiring ECMO implemented after the full-recommended 10-day dexamethasone course. Since these patients may have developed a particularly severe presentation, new therapeutic strategies are urgently required.

## 1. Introduction

Treatment of acute respiratory distress syndrome (ARDS) induced by coronavirus disease-2019 (COVID-19) relies on supportive intensive care, optimized mechanical ventilation and immunomodulatory treatments including a 10-day course of corticosteroids (based on the RECOVERY study) [1] and/or other immune modulators such as tocilizumab [2]. In extremely severe cases, profound hypoxemia and/or hypercapnia may require venovenous extracorporeal membrane oxygenation (ECMO) [3,4] while awaiting pulmonary recovery. In a large multicenter cohort study, survival of ECMO-treated COVID-19 patients was found to be ~46% [4]; but less than 30% of the selected patients received corticosteroids. Surprisingly, in other large published cohorts, corticosteroids were not even accounted for. Clinical trials supporting the benefit of corticosteroids included only a minority of COVID-19 patients treated with ECMO early in the clinical course. Interestingly, in the RECOVERY study, only 16% of the patients were mechanically ventilated and/or treated with ECMO at randomization [1].

Whether the same corticosteroid dose regimen and course duration could be as effective in extremely severe COVID-19 patients as in those without respiratory assistance remains unknown. Although corticosteroids improved overall prognosis, deteriorations were observed, leading to persistently high mortality rate. In patients developing refractory hypoxemia despite corticosteroids, ECMO was an option but survival probability remained unknown. 

This is an important issue as patients who deteriorate to the point of requiring ECMO despite the full-recommended 10-day corticosteroid course may present with a more severe disease and higher mortality. We therefore designed this study to investigate if ECMO implemented after corticosteroid failure can improve survival in COVID-19 patients.

## 2. Materials and Methods

### 2.1. Study Design and Purposes

We conducted a retrospective observational cohort study in three University hospitals located in French regions heavily impacted by the pandemic (Paris, Ile de France and Reims, Champagne-Ardennes). The study was performed according to the 2013 Declaration of Helsinki of the World Medical Association regarding medical investigations. It was part of the COVID-ICU and French COVID-19 cohort registries, approved by our institutional ethics committee (IDRCB, 2020-A00256-33; CPP, 11-2020.02.04.68737).

Our main purpose was to compare survival defined as discharge alive from hospital of critically ill COVID-19 patients in relation to corticosteroids and ECMO initiation timing. Survival was compared between patients who required ECMO for ARDS deterioration after a full 10-day corticosteroid course (*ECMO-after* group) and patients who required ECMO before the last corticosteroid dose on day 10 (*ECMO-before* group). Our secondary purpose was to identify factors associated with survival including the type of corticosteroid regimen and times from initial symptoms to corticosteroid initiation, from initial symptoms to ECMO implementation, and from intensive care unit (ICU) admission to ECMO implementation.

### 2.2. Study Population

All successive critically ill COVID-19 adults admitted to the three ICUs and treated with corticosteroids and venovenous ECMO were included. Patients who did not require venovenous ECMO during the ICU stay and patients treated with other types of extracorporeal assistance such as veno-arterial or veno-arterio-venous ECMO were not included.

COVID-19 patient management changed during the epidemic according to the available data supporting the effectiveness of proposed treatments. In both centers, dexamethasone was administered in COVID-19 patients with severe ARDS [5] since the beginning of the pandemic, using a dose regimen proposed in ARDS patients [6] consisting in an initial 20 mg/day dose for 5 days followed by a 10 mg/day dose for the 5 subsequent days. After publication of the RECOVERY trial results on dexamethasone (17 July 2020) [1], the administered dose regimen was changed to 6 mg/day for 10 days. Consequently, in the first months of the pandemic, patients received dexamethasone if ARDS was classified as severe according to the Berlin criteria6 after an initial evaluation excluding bacterial infection. After 17 July 2020, dexamethasone were systematically initiated on ICU admission. Patients admitted in the medical wards were also treated with the same regimen and transferred to the ICU if deteriorating despite dexamethasone. Other immunomodulatory therapies were administered at the discretion of physicians in charge.

### 2.3. Ventilation and ECMO Management in the ICU

Patients were first treated with high-flow oxygen, non-invasive ventilation or a combination of both. If pulmonary function deteriorated further, they received invasive mechanical ventilation with optimized positive end-expiratory pressure, ≤6 mL/kg tidal volume, ≤30 cm H_2_O or maximum 35 cm H_2_O plateau pressure if possible and permissive hypercapnia for arterial pH ≥ 7.25. Inspired oxygen fraction (FiO_2_) was adjusted to obtain PaO_2_ ≥ 60 mm Hg throughout the entire study period. Prone positioning was performed according to the usual criteria [7]. Nitric oxide and almitrine were administered for persistent hypoxemia according to physicians in charge. If severe hypoxemia and/or hypercapnia and/or respiratory acidosis persisted despite these interventions, ECMO was considered as rescue therapy. Indications of ECMO therapy were assessed by the ICU team, considering the degree of hypoxemia as suggested previously [8], respiratory acidosis, plateau pressure, advanced age, general health status, frailty, presence of debilitating disease and advanced directives if available. Thus, decision to implement ECMO in the present study was based on the usual clinical practice criteria and was not influenced by the corticosteroid treatment.

ECMO was implemented by ICU physicians using the Seldinger technique for vessel cannulation. After ECMO implementation, the patients received optimized supportive care and ventilator settings were adjusted to maintain PaO_2_ ≥ 60 mm Hg and PaCO_2_ in the normal range if possible, with high positive end-expiratory pressure, tidal volume of ≤6 mL/kg of ideal body weight, and respiratory rate of ≤20/min.

### 2.4. Data Collection

Data were collected retrospectively from electronic medical records. We collected the main demographic, laboratory, ventilation, corticosteroid and immunomodulatory treatment, and outcome data. Outcome was recorded as alive if the patient was discharged alive from hospital. The Sepsis-related Organ Failure Assessment (SOFA) score was calculated on admission [9]. In patients who required ECMO after a full 10-day dexamethasone course, a daily PaO_2_/FiO_2_ ratio was recorded from the first dose until ECMO implementation to describe its time-course pattern.

### 2.5. Statistical Analysis

Results are presented as medians (25th–75th percentiles) for quantitative parameters and percentages for qualitative parameters. Data were compared using Fischer’s exact-tests for categorical data and Mann–Whitney tests for numerical variables. PaO_2_/FiO_2_ ratios were compared using Friedman tests for paired samples. Parameters were compared between survivors versus non-survivors and univariate logistic regressions were performed to determine parameters associated with survival. Due to the limited sample size, a multivariable analysis of predictors of survival was not performed. Statistical analyses were performed using R statistical software version 3.6.3. Two-sided *p*-values < 0.05 were considered significant.

## 3. Results

### 3.1. Patient Characteristics

**Figure 1:**
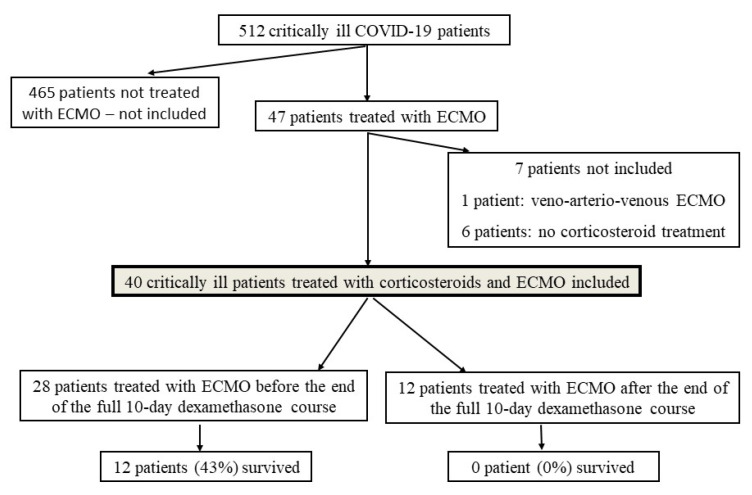
Chart flow.

During the study period, 512 critically ill COVID-19 patients were managed in the two ICUs including 47 patients treated with ECMO. Seven patients were not included in the study, since six of them did not receive any corticosteroid treatment and one was treated with veno-arterio-venous ECMO. Therefore, forty patients (28M/12F; age, 58 years (53–62); body mass index, 31 kg/m^2^ (27–35); past hypertension, 13/40 (36%); diabetes mellitus, 13/40 (36%)) were included (Figure 1), 28 (70%) in the ECMO-before group and 12 (30%) in the ECMO-after group. Patient characteristics are represented in Table 1.

Baseline parameters did not differ between the two groups except for the SOFA score on admission, which was lower in the ECMO-after group (5 (3–6) versus 9 (6–13), *p* = 0.02). There was a tendency to older age in the ECMO-after group (62 years (57–66) versus 57 years (51–59); *p* = 0.053). Laboratory parameters did not significantly differ between the two groups (Table 2).

### 3.2. Dexamethasone and Other Immunomodulatory Treatments

All patients received dexamethasone. The 6 mg/day dose regimen was administered to 9 (32%) in the ECMO-before group versus 12 (100%) in the ECMO-after group (*p* < 0.0001; Table 3). Dexamethasone was initiated 7 days (6–7) after the first symptoms in the ECMO-after group versus 12 days (8–15) in the ECMO-before group (*p* = 0.003). Tocilizumab was administered in eight patients and hydroxychloroquine/azithromycin in five patients.

### 3.3. ECMO Treatment and Correlates of Survival

PaO_2_/FiO_2_ ratio on optimized mechanical ventilation at ECMO initiation was 56 mmHg (48–66). Inhaled nitric oxide was administered at cannulation in 27 patients (75%) and almitrine in 15 patients (33%) without significant differences between groups. ECMO was initiated 3 days (1–5) and 7 days (2–11) after tracheal intubation in the ECMO-before versus ECMO-after group (*p* = 0.04). ECMO was initiated earlier during the clinical course in the ECMO-before compared to the ECMO-after group (12 days (10–14) versus 19 days (16–28) since the first symptoms, *p* = 0.0002; Table 3). ECMO treatment lasted for 18 days (14–26).

In the ECMO-before group, 12 (43%) survived while in the ECMO-after group, none (0%) survived (*p* = 0.007). Death cause was septic shock in 14 patients (35%), multiorgan failure without documented infection in 9 patients (23%), thrombosis or hemorrhage in 4 patients (10%) and persistent hypoxemia in one (2%). Based on univariate analyses, parameters significantly associated with survival were the time from ICU admission to ECMO, and time from intubation to ECMO, age, arterial pH at cannulation, plateau pressure and 6 mg/day dexamethasone regimen (Table 4 and Table 5).

### 3.4. Time-Course of the PaO_2_/FiO_2_ Ratio in the ECMO-after Group

**Figure 2:**
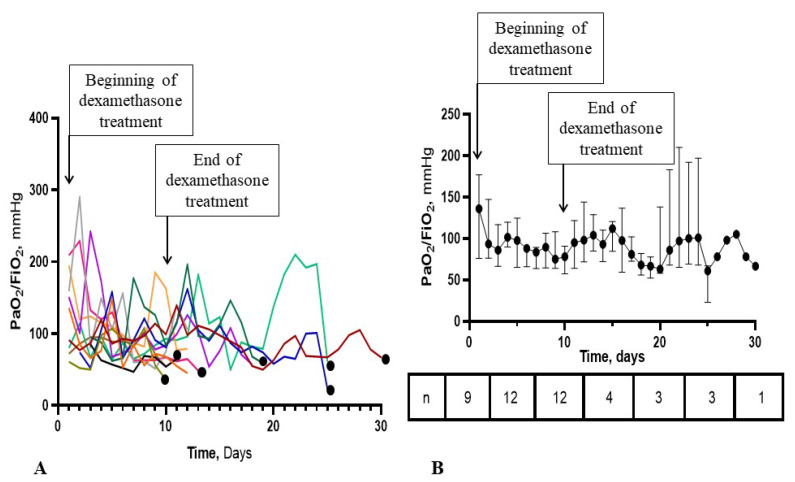
Individual and median PaO_2_/FiO_2_ ratios from corticosteroid initiation to ECMO implementation in the 12 patients included in the ECMO-after group. Panel (**A**) represents PaO_2_/FiO_2_ curves in individual patients. Data are missing in 3 patients on day 1. Curves end the day of ECMO implementation. Black dots identify patients with a diagnosis of bacterial pneumonia 48h before or after the day of ECMO initiation. Panel (**B**) represents median values and interquartile ranges as vertical bars. Numbers at the bottom represent the number of patients with available data. After day 10, the number of patients decreased as ECMO was initiated and only PaO_2_/FiO_2_ ratios in ECMO-free patients were represented. The beginning and the end of corticosteroid treatment are shown by the corresponding arrows in both panels.

**Figure 3:**
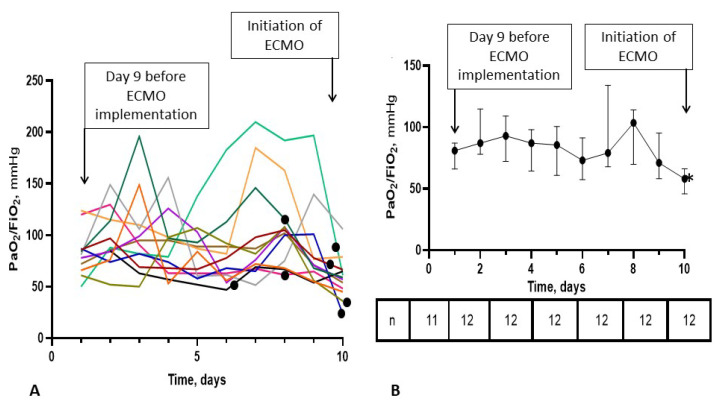
Individual and median PaO_2_/FiO_2_ ratios during the 10 days before ECMO implementation in the 12 patients included in the ECMO-after group. Panel (**A**) represents PaO_2_/FiO_2_ ratios of individual patients during the 9 days preceding ECMO initiation and the day of ECMO initiation (corresponding arrows). Data is missing in one patient on day 9 before ECMO initiation. Black dots in panel A represent patients with a diagnosis of bacterial pneumonia 48 h before or after the day of ECMO initiation. These were placed at different time points to avoid overlap with other curves. Panel (**B**) represents median PaO_2_/FiO_2_ ratios and interquartile ranges as vertical bars. * Significant difference in day10 (days of ECMO implementation) compared to day 2, 3, 4, 7, 8 by Freedman tests for paired samples.

The time-course of the PaO_2_/FiO_2_ ratio is shown in Figure 2 and Figure 3. In some patients, PaO_2_/FiO_2_ did not improve significantly until ECMO initiation while in others, the ratio increased to as high as 200 mmHg and decreased before ECMO initiation. On ECMO initiation, a significant decrease in PaO_2_/FiO_2_ ratio was observed but no such significant decrease was observed in the days preceding ECMO initiation (Figure 3). In seven patients (58%), positive bronchial aspirates were documented 48 h before or after ECMO implementation and may have been the reason for PaO_2_/FiO_2_ deterioration. Among the seven patients with positive bronchial aspirates, five required ECMO particularly late during the clinical course, day 4 or later after the end of dexamethasone treatment (Figure 2).

## 4. Discussion

We evaluated the survival rate at hospital discharge in patients requiring ECMO for aggravating ARDS during or after the recommended 10-day dexamethasone course in critically ill COVID-19 patients. The major finding of our study is that survival was null in patients in whom ECMO was required after the end of the full dexamethasone course (Figure 1). Although corticosteroid allowed a major improvement in COVID-19 patient outcome [10], management of patients who fail to improve under corticosteroids is still challenging, as shown in our study. In the *ECMO-after* group, PaO_2_/FiO_2_ ratio curves clearly showed no improvement in some patients despite 6 mg dexamethasone but marked improvement in others to values up to 200 mm Hg, followed by subsequent decrease as shown in Figure 2 and Figure 3. Our observations suggest that some patients did not respond to dexamethasone while others responded initially then deteriorated due to superinfections or COVID-19-related inflammation relapse. Thus, low PaO_2_/FiO_2_ ratios during and after the end of the corticosteroid course may herald a prolonged COVID-19 course and/or an incomplete response to corticosteroids, favoring hospital-related complications including superinfections. In most of our patients, once the recommended 10-day dexamethasone treatment ended, patients received essentially supportive care, close monitoring and treatment of complications. Despite effective treatment in patients with documented superinfections, pulmonary function did not improve and patients could not be weaned from ECMO. In the five patients without documented pulmonary infections before and after ECMO implementation, deterioration most likely occurred due to the underlying COVID-19. To date, no new treatment strategy has been proven useful in this setting. Some authors suggested that higher corticosteroid doses [11] and/or high-dose methylprednisolone boluses [12,13] may be effective as rescue therapy and that duration of treatment may have to be adapted to the disease length and severity, instead of being administered on a one size fits all basis [14]. Interestingly, a very recently published randomized control studies showed that 12 mg/day compared with 6 mg/day dexamethasone did not result in significantly more days alive without life support at 28 days among patients with COVID-19 and severe hypoxemia [15]. However, the authors acknowledged that the trial might have been underpowered. Therefore, it remains to be determined whether other immune modulators may be useful in these very severe patients. The dismal survival in the *ECMO-after* group also raises the question of futility of ECMO if no new therapeutic options become available, especially in periods of pandemic when healthcare resources become scarce.

Previous studies showed that age and shorter time from intubation to ECMO implementation are associated with survival [4], suggesting that beyond 7 days of intubation, survival is null [16]. In the *ECMO-after* group, 50% of our patients received ECMO earlier than 7 days after intubation. Therefore, we consider that increased mortality rate is not exclusively due to the time from intubation to ECMO implementation although this time may have contributed. In a large cohort, age and pH at cannulation were determined as major prognostic factors of survival [17]; but these parameters did not significantly differ in our study between the *ECMO-before* and *ECMO-after* groups. Interestingly, requiring ECMO after the end of the recommended full 10-day corticosteroid course was not associated with mortality in univariate analysis in our cohort. Therefore, this characteristic cannot be considered as a primary independent predictor of mortality in the overall population, but should be regarded as a major characteristic identifying a particularly severe patient subgroup with extremely high mortality.

In our patients, we reported an overall survival rate of 30% versus 46% in the study by Lebreton et al. [4]. Differences may be explained by an older age in our patients (58 years (53–62) versus 52 years (45–58)) and a lower PaO_2_/FiO_2_ ratio at cannulation (57 mm Hg (48–68) versus 61 mm Hg (54–70)) [4]. Moreover, the very high mortality in the *ECMO-after* group accounted for the high overall mortality while survival rate in the *ECMO-before* group was 43%, similar to the 46% rate noted in the study by Lebreton et al. [4]. Recently, based on the international Extracorporeal Life Support Organization Registry, mortality after ECMO for patients with COVID-19 was shown to have worsened during 2020 [18]. ECMO after 1 May 2020 had a higher likelihood of treatment-refractory disease despite similar risk factors, and centres with less experience providing ECMO support for COVID-19 patients were more likely to have a higher mortality rate. These observations clearly suggested that prognosis in ECMO-treated COVID-19 patients is multifactorial and does not only depend on patient-related criteria but on various patient-independent parameters related to the pandemic. Surprisingly, the relation between failure of ECMO rescue therapy and the duration of corticosteroid administered before ECMO implementation was not investigated in this registry-based study as we did here.

None of our patients achieved the conditions requested for lung transplantation by the thoracic surgeons of our university hospital group. Lung transplant in COVID-19 patients is a controversial issue, despite recently reported excellent results as option of last resort [19]. Whether some selected COVID-19 patients with end-stage lung disease might be candidates for lung transplant from compromised donors, remains also to be investigated.

Our study has limitations. Patients in the ECMO-before group were admitted late after symptom onsets (12 days (8–15)) in comparison to patients in the *ECMO-after* group (7 days (6–7); *p* = 0.003). The delay from intubation to ECMO was also significantly different between groups (3 days (1–5) versus 7 days (2–11); *p* = 0.04). Thus, we cannot assume that all characteristics of the two compared groups were the same, meaning that patients in the *ECMO-after* group may have missed the window of the optimal treatment opportunity explaining the worse survival in this group. However, at the ECMO implementation, no remarkable cardiac dysfunction nor differences in organ failure, vasopressor infusion and other treatment use were observed between the two groups. Interestingly, according to the SOFA score on admission, which significantly differed between the two patients groups, survival rate would have been expected to be lower in the *ECMO-before* than in the *ECMO-after* group, contrasting with what was observed (43% versus 0%, respectively). Therefore, our finding clearly suggests that ECMO requirement after the completion of the recommended full 10-day corticosteroid course identified a particularly severe patient group with extremely high mortality rate. The small sample size precluded any multivariate analysis to determine the factors independently associated with mortality. In univariate analyses, major prognostic factors known as prognosticators in COVID-19 patients requiring ECMO (age, arterial pH at ECMO implementation, time from intubation to ECMO implementation) were identified by our analysis as well as other factors, which are useful to generate hypotheses for future research, such as the negative correlation with survival of the time between ICU admission and ECMO implementation and of the 6 mg dexamethasone dose regimen. Thus, prolonged treatment with corticosteroids and/or higher doses may be tested in the future, in patients who require ECMO after the end of the initial ten-day corticosteroid course. Timing of ECMO implementation in our study may have been later than what was suggested, as PaO_2_/FiO_2_ of 80 mm Hg was put forth as cutoff [8], while our patients received ECMO at later stages, due to logistical issues related to the pandemic. Therefore, we cannot exclude that the outcome may have been different if the ECMO was initiated earlier in both groups, although there is no available randomized data supporting this hypothesis in COVID-19 patients or ARDS patients in general. For instance, although the fact that non-survivors appeared to be markedly older was predictable, we cannot exclude that age delayed decision of ECMO initiation due to resource rationing in the initial days of the pandemic. Finally, we identified a COVID-19 population with an extremely high risk of death despite management based on current knowledge on ARDS treatment including ECMO rescue therapy; however, we could not determine the exact causes of severity and the optimal therapeutic strategies to improve survival. Therefore, our study is a call for urgently needed research for new therapeutic strategies in COVID-19 patients with refractory ARDS.

## 5. Conclusions

Our study showed a dismal survival in COVID-19 patients who required ECMO after the end of the recommended 10-day dexamethasone course, which calls for new therapeutic strategies to improve outcome. Patients with advanced lung injury from COVID-19 may benefit from extreme supportive measures, such as ECMO, if considered early during the clinical course. Therefore, future studies that may help understanding the best ECMO management of these complex patients are needed.

## Figures and Tables

**Table 1:** Clinical characteristics on intensive care unit admission.

Parameters *	All Patients (N = 40)	ECMO before/during the 10-Day Dexamethasone Course (N = 28)	ECMO after the 10-Day Dexamethasone Course (N = 12)	*p*-Value
Age (years)	57 (51–62)	57 (51–59)	62 (57–67)	0.053
Male gender, N (%)	30 (75)	22 (79)	8 (67)	0.46
Body mass index (kg/m^2^)	31 (28–35)	32 (28–35)	30 (27–32)	0.38
History of hypertension, N (%)	16 (40)	9 (32)	7 (58)	0.17
Diabetes mellitus, N (%)	16 (40)	9 (32)	7 (58)	0.17
Ischemic heart disease, N (%)	3 (8)	2 (7)	1 (8)	1.0
History of heart failure, N (%)	1 (3)	1 (4)	0 (0)	1.0
Long-term anticoagulation, N (%)	2 (5)	1 (4)	1 (8)	0.52
Long-term aspirin treatment, N (%)	6 (15)	3 (11)	3 (25)	0.34
PaO_2_/FiO_2_ (mm Hg)	84 (71–136)	79 (61–111)	93 (76–158)	0.36
SOFA score on admission	8 (4–12)	9 (6–13)	5 (3–6)	0.02

* Data are expressed as median (25th–75th percentiles) or percentages. ECMO, extracorporeal membrane oxygenation; SOFA, Sepsis-related Organ Failure Assessment.

**Table 2:** Parameters at ECMO implementation.

Parameters *	All Patients (N = 40)	ECMO before/during the 10-Day Dexamethasone Course (N = 28)	ECMO after the 10-Day Dexamethasone Course (N = 12)	*p*-Value
Serum creatinine (µmol/L)	116 (64–265)	152 (71–283)	92 (61–139)	0.14
Serum alanine aminotransferase (IU/L)	38 (24–80)	38 (26–66)	44 (22–92)	0.95
Blood lactate (mmol/L)	1.5 (1.2–2.8)	1.5 (1.3–2.9)	1.6 (1.2–2.2)	0.81
C-reactive protein (mg/L)	155 (94–264)	155 (97–222)	177 (56–306)	0.83
Procalcitonin (µg/L)	1.40 (0.2–3.14)	1.25 (0.62–2.85)	2.19 (0.19–5.49)	0.95
Fibrinogen (g/L)	6.0 (4.8–7.6)	5.8 (4.4–7.1)	6.3 (5.4–8.9)	0.18
D-dimer (ng/mL)	3195 (1858–6208)	2940 (1735–3550)	5750 (2975–16,310)	0.09
PaO_2_/FiO_2_ (mm Hg)	56 (48–66)	55 (48–66)	56 (53–67)	0.58
PaCO_2_ (mmHg)	58 (47–67)	56 (45–65)	59 (48–69)	0.55
Arterial pH	7.31 (7.23–7.37)	7.32 (7.25–7.38)	7.27 (7.20–7.34)	0.23
Shock, N (%)	36 (90)	25 (89)	11 (92)	1.0
Renal replacement therapy, N (%)	20 (50)	14 (50)	6 (50)	1.0
Time from first symptoms to ECMO (days)	13 (10–17)	12 (10–14)	19 (16–28)	0.0002
Length of ICU stay (days)	31 (19–46)	30 (17–46)	37 (22–45)	0.42
Length of hospital stay (days)	39 (22–59)	37 (21–62)	39 (25–47)	0.64

* Data are expressed as median (percentiles 25th–75th) or percentages. ECMO, extracorporeal membrane oxygenation; ICU, intensive care unit.

**Table 3:** Survival and immunomodulatory drugs, mechanical ventilation, and ECMO.

Parameters *	All Patients (N = 40)	ECMO before/during the 10-Day Dexamethasone Course (N = 28)	ECMO after the 10-Day Dexamethasone Course (N = 12)	*p*-Value
Survival, N (%)	12 (30)	12 (43)	0 (0)	0.007
Dexamethasone treatment, N (%)	40 (100)	28 (100)	12 (100)	1.0
Dexamethasone 6 mg/day regimen, N (%)	21 (53)	9 (32)	12 (100)	<0.0001
Dexamethasone 20/1 mg/day regimen, N (%)	19 (47)	19 (78)	0 (0)	<0.0001
Time from first symptoms to dexamethasone (days)	9 (7–13)	12 (8–15)	7 (6–7)	0.003
Tocilizumab treatment, N (%)	8 (20)	4 (14)	4 (33)	0.21
Number of prone sessions before ECMO	3 (2–4)	3 (2–4)	3 (2–5)	0.75
Inspired tidal volume (mL)	352 (335–406)	360 (343–400)	350 (300–391)	0.49
Plateau pressure (cm H_2_O)	30 (28–32)	29 (25–30)	32 (29–34)	0.013
Positive end-expiratory pressure (cm H_2_O)	12 (10–14)	12 (10–14)	12 (10–14)	0.65
Static compliance (mL/cm H_2_O)	22 (16–29)	25 (21–30)	16 (13–19)	0.02
Respiratory rate (cycle/min)	30 (28–34)	29 (27–32)	33 (32–35)	0.03
Total duration of mechanical ventilation (days)	28 (16–35)	26 (16–36)	30 (19–36)	0.82
Time from ICU admission to ECMO (days)	7 (4–9)	5 (2–7)	12 (10–19)	<0.0001
Time from intubation to ECMO (days)	4 (1–6)	3 (1–5)	7 (2–11)	0.04
ECMO duration (days)	14 (7–26)	13 (6–21)	26 (11–31)	0.20

* Data are expressed as median (25th–75th percentiles) or percentages. ECMO, extracorporeal membrane oxygenation; ICU, intensive care unit.

**Table 4:** Comparison of main characteristics between survivors and non-survivors.

Parameters *	Survivors (N = 12)	Non-Survivors (N = 28)	*p*-Value
Age (years)	51 (43–56)	60 (57–64)	0.0006
SOFA score on admission	9 (4–11)	7 (5–12)	0.84
Time from first symptom to ECMO (days)	12 (12–14)	14 (10–17)	0.31
Time from ICU admission to ECMO (days)	4 (2–6)	7 (5–11)	0.017
Time from intubation to ECMO (days)	3 (0–4)	5 (2–7)	0.03
Time from first symptom to dexamethasone (days)	13 (7–15)	9 (6–10)	0.07
Serum creatinine (µmol/L)	112 (54–196)	116 (78–272)	0.53
Renal replacement therapy, N (%)	3 (25)	17 (61)	0.08
Blood lactate (mmol/L)	1.4 (1.2–2.0)	1.6 (1.2–2.8)	0.56
PaO_2_/FiO_2_ at cannulation (mm Hg)	55 (45–61)	56 (48–68)	0.43
Presence of shock, N (%)	10 (83)	26 (93)	0.57
Arterial pH at cannulation	7.37 (7.34–7.45)	7.27 (7.20–7.33)	0.002
Plateau pressure (cmH_2_O)	26 (21–30)	30 (29–33)	0.015
Static compliance (ml/cmH_2_O)	27 (20–30)	21 (15–25)	0.11
Positive end-expiratory pressure (cmH_2_O)	12 (10–14)	12 (10–14)	0.95
Dexamethasone 6mg/day regimen, N (%)	3 (25)	18 (64)	0.038
ECMO after at least 10-day dexamethasone, N (%)	0 (0)	12 (43)	0.007

* Data are expressed as median (percentiles 25th–75th) or percentages. ECMO, extracorporeal membrane oxygenation; ICU, intensive care unit.

**Table 5:** Parameters associated with survival in univariate analyses.

Parameters	Odds Ratio (CI) *	Area under the Curve of the Model (CI)	*p*-Value
Age	0.855 (0.764–0.957)	0.844 (0.694–0.939)	0.0005
pH at cannulation	1.97 × 10^6^ (44.600–87.3 × 10^9^)	0.854 (0.696–0.949)	0.0008
Time from ICU admission to ECMO	0.796 (0.642–0.986)	0.740 (0.577–0.865)	0.007
Plateau pressure	0.762 (0.601–0.951)	0.786 (0.606–0.911)	0.005
Time from intubation to ECMO	0.759 (0.575–1.002)	0.717 (0.553–0.848)	0.019
Dexamethasone 6mg/day regimen	0.1852 (0.041–0.845)	0.696 (0.531–0.832)	0.021

* An odds ratio > 1 represents a positive association while an odds ratio < 1 a negative association between a variable and survival. CI, 95%-confidence interval; ECMO, extracorporeal membrane oxygenation; ICU, intensive care unit.

## Data Availability

Mégarbane have full access to all data and takes responsibility for the data integrity and its analysis accuracy. Data supporting reported results can be obtained from the corresponding author if reasonably justified.
